# How frequently should “living” guidelines be updated? Insights from the Australian Living Stroke Guidelines

**DOI:** 10.1186/s12961-022-00866-7

**Published:** 2022-06-20

**Authors:** Tari Turner, Steve McDonald, Louise Wiles, Coralie English, Kelvin Hill

**Affiliations:** 1 Cochrane Australia, School of Public Health and Preventive Medicine, Monash University, Level 4 553 St Kilda Road, Melbourne, VIC 3004 Australia; 2 IIMPACT in Health, UniSA Allied Health & Human Performance, The University of South Australia, Adelaide, Australia; 3 Australian Institute of Health Innovation, Macquarie University, Sydney, Australia; 4 South Australian Health and Medical Research Institute, Adelaide, South Australia; 5 School of Health Sciences, and Priority Research Centre for Stroke and Brain Injury, The University of Newcastle, Newcastle, Australia; 6 Stroke Foundation, Melbourne, Australia

**Keywords:** Guidelines, Evidence, Stroke, Living guidelines, Updating

## Abstract

**Background:**

“Living guidelines” are guidelines which are continually kept up to date as new evidence emerges. Living guideline methods are evolving. The aim of this study was to determine how frequently searches for new evidence should be undertaken for the Australian Living Stroke Guidelines.

**Methods:**

Members of the Living Stroke Guidelines Development Group were invited to complete an online survey. Participants nominated one or more recommendation topics from the Living Stroke Guidelines with which they had been involved and answered questions about that topic, assessing whether it met criteria for living evidence synthesis, and how frequently searches for new evidence should be undertaken and why. For each topic we also determined how many studies had been assessed and included, and whether recommendations had been changed.

**Results:**

Fifty-seven assessments were received from 33 respondents, covering half of the 88 guideline topic areas. Nearly all assessments (49, 86%) were that the continual updating process should be maintained. Only three assessments (5%) deemed that searches should be conducted monthly; 3-monthly (14, 25%), 6-monthly (13, 23%) and yearly (17, 30%) searches were far more frequently recommended. Rarely (9, 16%) were topics deemed to meet all three criteria for living review. The vast majority of assessments (45, 79%) deemed the topic a priority for decision-making. Nearly half indicated that there was uncertainty in the available evidence or that new evidence was likely to be available soon.

Since 2017, all but four of the assessed topic areas have had additional studies included in the evidence summary. For eight topics, there have been changes in recommendations, and revisions are underway for an additional six topics.

Clinical importance was the most common reason given for why continual evidence surveillance should be undertaken. Workload for reviewers was a concern, particularly for topics where there is a steady flow of publication of small trials.

**Conclusions:**

Our study found that participants felt that the vast majority of topics assessed in the Living Stroke Guidelines should be continually updated. However, only a fifth of topic areas were assessed as conclusively meeting all three criteria for living review, and the definition of “continual” differed widely. This work has informed decisions about search frequency for the Living Stroke Guidelines and form the basis of further research on methods for frequent updating of guidelines.

## Background

Living guidelines are guidelines which are continually kept up to date as new evidence emerges, with frequent searches for and incorporation of new research [1]. Importantly, living guidelines maintain the rigour of traditional evidence-based guideline development methods, and ensure that recommendations are reviewed and updated within months rather than years as traditionally has been the case [1].

The criteria to determine when a living, continual mode of updating is appropriate for a particular clinical topic area include the following:

1. The topic is an important priority for health decision-making; perhaps due to large numbers of people affected, substantial potential impact of the decision on health outcomes, or known variation in practice.
2. There is uncertainty in the existing evidence; for example, the certainty of the evidence is not high [2], or there is a lack of high-quality reviews; there are gaps in primary evidence or aspects of the topic not covered by the existing evidence (new interventions, subgroups, outcomes, etc.).
3. There is likely to be emerging evidence that will impact on the conclusions of the evidence synthesis, potentially leading to changes in recommendations; areas where new trials have been recorded in trials registries or where there is an ongoing, high rate of trial publication [3].

An additional consideration is whether there are adequate resources to conduct and screen the searches and update the evidence summaries.

Living guideline methods are in early development, and pilot projects are underway globally to produce living guidelines in areas including COVID-19, diabetes, arthritis and maternal health [4–7]. Established in 2018, the Stroke Foundation’s Clinical Guidelines for Stroke Management were one of the world’s first guidelines to implement continual updating methods.

One area of ongoing methods development in living guidelines is establishing the frequency with which recommendations need to be updated. Decisions about frequency have important implications for the resources required to conduct the searches, assess the impact of any new studies and incorporate new evidence. For other types of living syntheses, such as living systematic reviews, monthly searches are recommended [8].

The optimal frequency is likely to vary between topics depending on the urgency or importance of the issue and how rapidly the evidence base is evolving, as well as pragmatic issues like resourcing. For example, the Australian guidelines for the clinical care of people with COVID-19 are updated weekly; however, other living guidelines have been updated much less frequently, with searches undertaken monthly or quarterly [6, 7, 9]. At the commencement of the Living Stroke Guidelines, the project executive decided to test keeping the whole stroke guideline “living” rather than applying criteria to each topic, and to conduct monthly evidence surveillance to identify new evidence relevant to all the guideline recommendations.

As part of the process of reflecting on the methods used in the Living Stroke Guidelines, we undertook a survey of the Living Stroke Guidelines Development Group members to ascertain their views about the clinical topics they had been involved in for the Living Stroke Guidelines. To inform methods for the Living Stroke Guidelines, and also living guidelines methods more broadly, we aimed to explore whether Living Stroke Guidelines Development Group members felt these clinical topics met the three living evidence synthesis criteria [3], and how frequently they believed searches for new evidence should be undertaken.

## Methods

The Living Stroke Guidelines consist of eight chapters addressing 88 topic areas, for which nearly 300 recommendations are made [10]. Each topic is framed to address an aspect of care, for example the use of antiplatelet therapy for secondary prevention of stroke. Small working groups of clinical experts (three to five members) contribute to reviewing new evidence and proposing changes to recommendations as needed. Individual clinical experts are involved in one to five topic areas, with wider group member input (including consumers) provided to the proposed changes before sign-off by a multidisciplinary steering group.

For this research, there were two main data sources: (1) an anonymous online survey, and (2) evidence surveillance records for the living guidelines (i.e. studies assessed and included, recommendations changed). Clinical experts who are contributing to developing and maintaining the Living Stroke Guidelines were invited by email to participate in an anonymous, online survey conducted using Qualtrics^XM^. The survey was open for a 3-week period and asked participants to nominate one or more clinical topics from the Living Stroke Guidelines with which they had been involved and then to answer a series of questions about each topic area (see Box 1). Potential participants were emailed two reminders to participate.

Participants could elect to answer these questions for up to five topic areas they had been involved in. The survey included one additional free-text question not linked to any topic area: “What else should we consider in thinking about frequency of updating the Stroke Guidelines?”

Simple descriptive statistical analysis of the quantitative data was undertaken using Microsoft Excel. Qualitative data were imported into NVivo 12 and analysed using crafted codes which were then collapsed into key themes. Qualitative data were analysed by one author (TT) who is experienced in mixed-methods research and was involved in the initial development of the guideline methods, but who had no prior contact with the participants or involvement in the day-to-day operation of the guideline program.

To determine whether and how new research had influenced guideline recommendations in the period being examined, for each of the topic areas nominated by the participants, we reviewed our records to establish whether additional relevant research evidence had been identified by our evidence surveillance and incorporated into the guidelines since the guideline update in 2017 (prior to introduction of living methods), and whether the recommendation had changed during that time period.

Ethics approval was granted by the Monash University Human Research Ethics Committee (Project Number 26354). All participants provided informed consent.

Box 1 Survey question for each topic area

1. Should this topic area be continually updated, i.e. “living”? (Yes/No/Maybe)
*Is this topic a priority for decision-making? (Yes/No)
*Is there uncertainty in the currently available evidence for this topic? (Yes/No/Varies for specific interventions/outcomes)
*Is it likely that new evidence which may impact the guideline recommendations for this topic will become available soon? (Yes/No/Unsure)
5. How often do you think evidence for this topic should be searched for, reviewed and updated? (Monthly/Every 3 months/Every 6 months/Yearly/When a new update of an existing high-quality systematic review is published/Other)
6. Are there other considerations which might influence how often this topic should be updated? (Free text)
*These three questions are the criteria for appropriateness for living evidence synthesis approaches [3].

## Results

One hundred and two clinical experts in the Guidelines Group were invited to participate, and 33 participants contributed to this analysis (32% response rate; one response did not provide topic area names).

### Quantitative data

We received a total of 57 assessments, covering 44 unique topic areas (out of 88 addressed by the guidelines; see Table 1). Two participants assessed five topics, two addressed four topics, one addressed three topics, eight addressed two topics and 20 addressed one topic. Thirty-two topics were assessed once, 11 were assessed twice, one (walking) was assessed three times (see Table 1).

**Table 1:** List of topics assessed

Topic no.	Topic description	Number of assessments in survey	Number of studies assessed	Number of additional studies included	Recommendation changed (y/n)
*Met all criteria for living review according to both assessors*					
16	Early mobilization	2	15	5	No
*Met all criteria for living review according to the single assessor*					
20	Faecal incontinence	1	4	1	Update underway
22	Fatigue	1	12	6	No
24	Goal-setting	1	4	0	No
27	Memory	1	25	14	Update underway
36	Return to work	1	8	3	No
43	Treatment for depression and/or anxiety	1	88	43	Update underway
*Met all criteria for living review according to one of the multiple assessors*					
44	Walking	3	90	44	Yes
32*	*Pre-hospital care*	2	8	2	No
*Not assessed as a priority for decision-making by the single assessor*					
12	Contracture	1	8	2	No
13	Dysarthria	1	7	4	No
18	Early supported discharge services	1	5	2	No
28	Oedema/limb swelling	1	3	3	Update underway
23	Glucose management	1	13	3	No
33	Pyrexia management	1	3	2	No
34	Rehabilitation—cognitive communication deficits	1	1	0	No
38	Sexuality	1	1	1	No
40	Spasticity	1	104	41	No
*Not assessed as a priority for decision-making by one of the two assessors*					
11	Continence (urinary)	2	8	3	No
29	Oral health	2	10	5	Yes
32*	*Pre-hospital care*	2	8	2	No
25	Information and education	1	15	5	No
26	Loss of sensation	1	23	8	No
30	Palliative care	1	0	0	No
31	Patent foramen ovale	1	32	5	Yes
37	Secondary prevention^c^	2			
39	Shoulder pain	1	29	20	Yes
41	Standing	1	68	15	Update underway
42	Thrombolysis	1	54	10	Yes
1	Adherence to pharmacotherapy	1	45	36	No
2	Activities of daily living	1	49	16	Update underway
3	Amount of rehabilitation	2	1	1	No
4	Anticoagulant therapy	1	14	3	No
5	Antiplatelet therapy^a^	2	60	13	Yes
6	Blood pressure-lowering therapy^a^	2	53	12	No
7	Cardiorespiratory fitness training	2	40	15	No
8	Cholesterol-lowering therapy	1	16	10	Yes
9	Clot retrieval	2	85	4	No
10	Comprehensive swallowing assessment	1	29	3	No
14	Dysphagia screening	1	31	6	Yes
15	Early hydration	1	6	0	No
17	Early nutrition	1	6	3	No
19	Executive function post stroke	1	17	11	No
35	Resource use^b^	1			
21	Falls	2	27	15	No

^a^Includes both immediate and long-term therapy. ^b^Unclear topic mapping. ^c^This is a new topic, in development at time of data collection

*This topic is in multiple rows as it received two assessments which differed

#### Should this topic area be continually updated, i.e. “living”?

Participants reported that the vast majority of topic areas assessed should be continually updated [yes (49, 86%), maybe (5, 9%), no (3, 5%)].

The eight topics reported as not, or only potentially, needing to be continually updated are provided in Table 2 along with the assessment against each of the three living review criteria. One topic (return to work) was rated as meeting all three criteria for living review, in spite of not being assessed as needing continual update. Two topics (contracture and spasticity) were rated as meeting none of the three criteria.

**Table 2:** Topic areas assessed as “should NOT be continually updated” or “should POSSIBLY be continually updated”

	Living review criteria		
Topic	1. Priority for decision-making	2. Uncertainty in current evidence	3. Likely that new evidence will emerge
*Should not be continually updated*			
Contracture	✗	✗	✗
Oral health	✗	✓	✓
Palliative care	✓	✓	✗
*Should possibly be continually updated*			
Return to work	✓	✓	✓
Amount of rehabilitation	✓	✓	✗
Dysarthria	✗	✓	✗
Sexuality	✗	✓	✗
Spasticity	✗	✗	✗

#### Does this topic meet the living evidence synthesis criteria?

Ten assessments, of nine topics, were that topics met all three criteria for living review (identified by ^ in Table 1) by at least one assessor. Early mobilization was rated as meeting all criteria for living review by two assessors. For pre-hospital care and walking, at least one assessor rated the topic as meeting all three criteria.

Forty-five assessments (79%) indicated the topic was a priority for decision-making. Three topics were assessed by two participants, and there was disagreement between assessors as to whether they were a priority for decision-making.

Twenty-eight assessments (49%) reported uncertainty in the available evidence; for 21 (37%), this varied with specific interventions or outcomes, and for eight (14%), there was not felt to be uncertainty.

There was a similar pattern for likely future availability of evidence, with 26 (46%) assessments that new evidence was likely, 24 (42%) unsure and seven (12%) that new evidence was unlikely to be available soon.

#### How often do you think evidence for this topic should be searched for, reviewed and updated?

For suggested frequency of updating, three assessments (5%) deemed this should be monthly, 14 (25%) every 3 months, 13 (23%) every 6 months, 17 (30%) yearly, four (7%) when a new update of a high-quality systematic review is published, and six (11%) other, with a variety of suggestions made, mostly linked to publication of large trials.

### Data from evidence surveillance

Data on results of searches and impact on guideline recommendations are provided in Table 1. Since the guidelines transitioned to continual updating in 2017, all but one of the 44 assessed topic areas (palliative care) have had new studies assessed for inclusion, and all but four topic areas (palliative care, cognitive communication deficits, goal-setting, and early hydration) have had additional studies included in the guideline evidence summary. For eight topic areas, these new studies have led to changes in recommendations, and for an additional six topic areas, revisions to recommendations are currently underway. For almost two thirds of topics, no change has been made to the relevant recommendations.

### Qualitative data

The *importance* of the topic area was the most common reason given for why continual evidence surveillance should be undertaken. Topic areas across the scope of the guideline were described as “*critical*” [ID 29], “*the most important topic*” [ID 38] or “*very important topic*” [ID 35].

Respondents also highlighted the need to ensure that consumers were consulted in the process of assessing the importance of topic areas, noting that “*it would be good to get consumer input into these questions as well as the stroke team. We do not always fully appreciate what topics are important in recovery beyond the main medical and functional considerations*” [ID 35].

The *likelihood that new evidence would change the recommendation* was also felt to be an important determinant of how often new evidence should be incorporated. Respondents noted that for some topic areas, including every new trial led to “*lots of work for little relative change to recommendations*” [ID 14], and that for these topics, updating “*more than yearly is not worth the resources*” [ID 38].

*Workload for reviewers* was frequently mentioned as a concern, particularly for topics where there is a large number of reviews of smaller trials. Recommendations on how to best manage this varied though, with some participants suggesting less frequent review, and others that frequent, smaller amounts of review work were more manageable.

*[This recommendation] is likely to continue to be based on a large number of reviews of smaller trials. The time taken to go through this it makes sense to keep it to an annual review.* [ID 38]

*There is an overwhelming amount of evidence continually being produced for this topic. Because of this I think regular review of the evidence will make the workload more manageable.* [ID 4]

*Availability of new evidence* was highlighted as a key reason to update recommendations, and particularly publication of new, large randomized controlled trials. This was felt to be particularly important if the existing evidence base was mostly indirect evidence (e.g. from countries with differing health systems) or only addressed the practice of some clinical groups (e.g. physiotherapy but not nursing).

Two other issues related to *searching and evidence surveillance* were raised. One was a suggestion that only “*critical topics [that] are constantly under research (e.g. thrombolysis) be continually updated; and that, for other topics, a prompt system be set up to ask the guideline group members, perhaps on a quarterly basis, whether a recommendation need be updated*” [ID 29]. The second was that the team consider “*setting restrictions on the types of new papers that will pass initial screening (e.g. new RCTs and IPD [individual patient data] meta-analyses only for topics with well-established evidence)*” [ID 17].

Improvements to *communication* were also requested, particularly to clarify the role of the working groups and to share “*upcoming evidence*” [ID 33] to be presented at conferences or news of recently registered trials, which might lead to relevant results in the future.

## Discussion

Our study invited participants to assess the extent to which a continual model of updating was appropriate for the 88 topics covered by the Australian Living Stroke Guidelines. For the 44 topics assessed by the participants, the vast majority were deemed appropriate for continual updating. While limited by the topics for which responses we received, this data provides initial support of the decision to make the entire guideline, rather than selected recommendations, living. However, a key finding was the understanding of what would constitute “continual” update differed widely among the survey participants. Even when restricted to the topics which participants assessed as needing to be continually updated, the majority of assessments were that the evidence should be updated either six-monthly (25%) or yearly (29%)—a monthly search frequency was deemed necessary for only 6% of topic areas assessed. The qualitative data suggest that the assessment of updating frequency is likely to have been influenced by the perceived workload required to review the literature. Interestingly, there was a difference of opinion over whether less frequent but larger rounds of updating were likely to be more manageable than more frequent, smaller rounds of updating. These findings that appropriate frequency of updating is likely to vary with topic, align with those found by researchers working with standard guideline methods [11].

While most topics were assessed as needing continual updating, only nine topics conclusively met all three criteria for living review. The priority for decision-making criterion was met by more than three quarters of topic assessments; participants were less definite in their responses to the questions about the *uncertainty of existing evidence* and *likelihood of new evidence* criteria, with almost half giving equivocal responses for both questions. The central role of priority for decision-making as a criterion is strengthened by the similar results found in qualitative data. This raises the question of whether the assessment of appropriateness of living evidence synthesis might be improved if it was made jointly by teams with both the clinical expertise to assess priority for decision-making and the methodological skills to assess uncertainty and likely future flow of evidence. Alternatively, it might be that the priority for decision-making criterion should be more heavily weighted in the assessment of appropriateness of living evidence approaches.

The data from our evidence surveillance show that while new potentially relevant evidence was found for almost all of the topic areas assessed, in only a minority of cases did this new evidence lead to changes to recommendations. At the time of publication, new or updated recommendations were made to 16/44 topics included in this survey. Six of the remaining topics not coved by responses have been changed. This suggests that new evidence most frequently serves to bolster rather than challenge the results of existing evidence syntheses. The addition of new evidence which does not change the direction of recommendations appears to improve the perceived credibility and trustworthiness of the guidelines from end users’ perspectives [9]. An evaluation of this is underway.

As a pioneering project, the Living Stroke Guidelines are leading the way in exploring methodological issues for living guidelines. It will be valuable to compare experiences across the other living guidelines projects to determine variation in guideline topic and development approaches. It will also be useful to consider how methods developments in other areas of living evidence synthesis might or might not apply to living guidelines. For example, while the guidance for Cochrane living systematic reviews sets an expectation of monthly evidence searches [8], an evaluation of the feasibility and acceptability of living reviews highlighted concerns about the frequency of searching [12]. This echoes similar concerns expressed by the survey participants in this study. In practice, some living reviews are undertaking less frequent searches. Our results suggest less frequent searches may also be appropriate for some living guidelines.

The development of living guideline methods is in its infancy. This study provides useful insights into the perceptions of clinicians and guideline development group members on how decisions about frequency of updating living recommendations could or should be made. While there was only a reasonably small number of respondents (33), this represents a third of the 102 clinicians involved in the guidelines, and assessments were made of half of the topics covered by the guidelines. Topics not covered in responses to the survey included more acute medical areas, and fewer topics (6/44) had changes to the recommendations compared with those included in survey responses (16/33). It is unclear if this would significantly alter the current study outcomes. The study is also limited by our, largely pragmatic, decision to have assessments made at the level of the guideline topic rather than the specific question or recommendation. This means our data are not granular enough to identify variations within a topic in updating requirements; however, as an early investigation of this important topic, we feel it nevertheless makes a useful contribution. None of the participants expressed any concerns that their responses might not apply to the whole topic area.

The results of this study were used to refine methods for the Living Stroke Guidelines, with evidence surveillance continuing monthly but with input from clinical experts sorted every 6 months to confirm potential inclusion and impact. This reflects the clear opinion of the vast majority of responses that surveillance methods should continue for all topics, but that the frequency of clinical expert input is less than initially tested. However, evidence with a clear impact on existing recommendations, as judged by a central project team, will be fast-tracked for clinical expert input. Future work in this area should build on research undertaken to investigate definitions and methods for frequent updating of guidelines using traditional guideline development models [13] and the growing global experience of living guideline development. It will be particularly important to investigate how approaches vary between guideline topics.

## Conclusion

In this survey, members of the Living Stroke Guidelines Development Group considered that the majority of topic areas should be continually updated, or “living”, and that the definition of “continual” might include options in which searches for new evidence are undertaken on a 3- or 6-monthly basis, or less frequently for some recommendations. This research provides important insights into the application, and need for further development, of methods from a pioneering living guideline project.

## Data Availability

Data can be made available on reasonable request.

## References

[1] Akl EA, Meerpohl JJ, Elliott J, Kahale LA, Schunemann HJ, Living Systematic Review N (2017). Living systematic reviews: 4. Living guideline recommendations. J Clin Epidemiol..

[2] Alonso-Coello P, Schünemann HJ, Moberg J, Brignardello-Petersen R, Akl EA, Davoli M (2016). GRADE Evidence to Decision (EtD) frameworks: a systematic and transparent approach to making well informed healthcare choices. 1: Introduction. BMJ.

[3] Elliott JH, Synnot A, Turner T, Simmonds M, Akl EA, McDonald S (2017). Living systematic review: 1. Introduction-the why, what, when, and how. J Clin Epidemiol..

[4] Lamontagne F, Agoritsas T, Macdonald H, Leo YS, Diaz J, Agarwal A (2020). A living WHO guideline on drugs for covid-19. BMJ.

[5] Vogel JP, Dowswell T, Lewin S, Bonet M, Hampson L, Kellie F (2019). Developing and applying a 'living guidelines' approach to WHO recommendations on maternal and perinatal health. BMJ Glob Health.

[6] White H, Tendal B, Elliott J, Turner T, Andrikopoulos S, Zoungas S (2020). Breathing life into Australian diabetes clinical guidelines. Med J Aust.

[7] Tendal B, Vogel J, McDonald S, Norris S, Cumpston M, White H (2020). Weekly updates of national living evidence-based guidelines: methods for the Australian Living Guidelines for Care of People with COVID-19. J Clin Epidemiol.

[8] Brooker J, Synnot A, McDonald S, Elliott J, Turner T. Guidance for the production and publication of Cochrane living systematic reviews: Cochrane reviews in living mode. Version December 2019. 2019. https://community.cochrane.org/review-production/production-resources/living-systematic-reviews. Accessed 13 June 2022.

[9] Hill K, English C, Campbell BCV, McDonald S, Pattuwage L, Bates P (2022). Feasibility of national living guideline methods: the Australian Stroke Guidelines. J Clin Epidemiol.

[10] Clinical guidelines for stroke management: Stroke Foundation; 2021. https://informme.org.au/en/Guidelines/Clinical-Guidelines-for-Stroke-Management. Accessed 13 June 2022.

[11] Becker M, Neugebauer EA, Eikermann M (2014). Partial updating of clinical practice guidelines often makes more sense than full updating: a systematic review on methods and the development of an updating procedure. J Clin Epidemiol.

[12] Millard T, Synnot A, Elliott J, Green S, McDonald S, Turner T (2019). Feasibility and acceptability of living systematic reviews: results from a mixed-methods evaluation. Syst Rev.

[13] Martinez Garcia L, Pardo-Hernandez H, Sanabria AJ, Alonso-Coello P, Penman K, McFarlane E (2018). Guideline on terminology and definitions of updating clinical guidelines: The Updating Glossary. J Clin Epidemiol.

